# “Scientific or democratic?” Bridging the implementation gap of citizen science through the study of social-symbolic objects

**DOI:** 10.3389/fpubh.2026.1702812

**Published:** 2026-05-14

**Authors:** Jonathan Broekhuizen, Jouke De Jong

**Affiliations:** 1 Coordination of Urban Issues Research Group, Faculty of Applied Social Sciences and Law, Amsterdam University of Applied Sciences, Amsterdam, Netherlands; 2 Department of Health Sciences, Faculty of Sciences, Vrije Universiteit Amsterdam, Amsterdam, Netherlands

**Keywords:** assumptions, community-based participatory research, epistemology, normativity, problematization, quality criteria, relevance, rigor

## Introduction

Despite its promise, citizen science in public health is falling short of its potential. It is commonly framed as involving citizens as co-researchers—engaged across the full research process, from problem definition and study design to data collection and analysis, interpretation, and application. Yet the literature consistently points to a persistent implementation gap, variously described as a “rhetoric–reality gap” (1), a disconnect between principles and practice (2), or a case of rhetoric outpacing practice (3). Taken together, these critiques highlight a recurring concern: that citizen science is prone to tokenism—participatory in intention, but in practice often reducing citizens' roles to data collection rather than enabling meaningful involvement throughout the research process (4, 5). To bridge the gap, we argue that citizen science needs to reconcile two different and currently conflicting aims – achieving scientific rigor and democratic participation – and that this requires a rethinking of the role of participation in citizen science. We introduce the concept of social-symbolic objects as a way to understand how genuine participation across all research stages may not only avoid impeding but in fact promote rigor and credibility in public health research.

**Figure 1:**
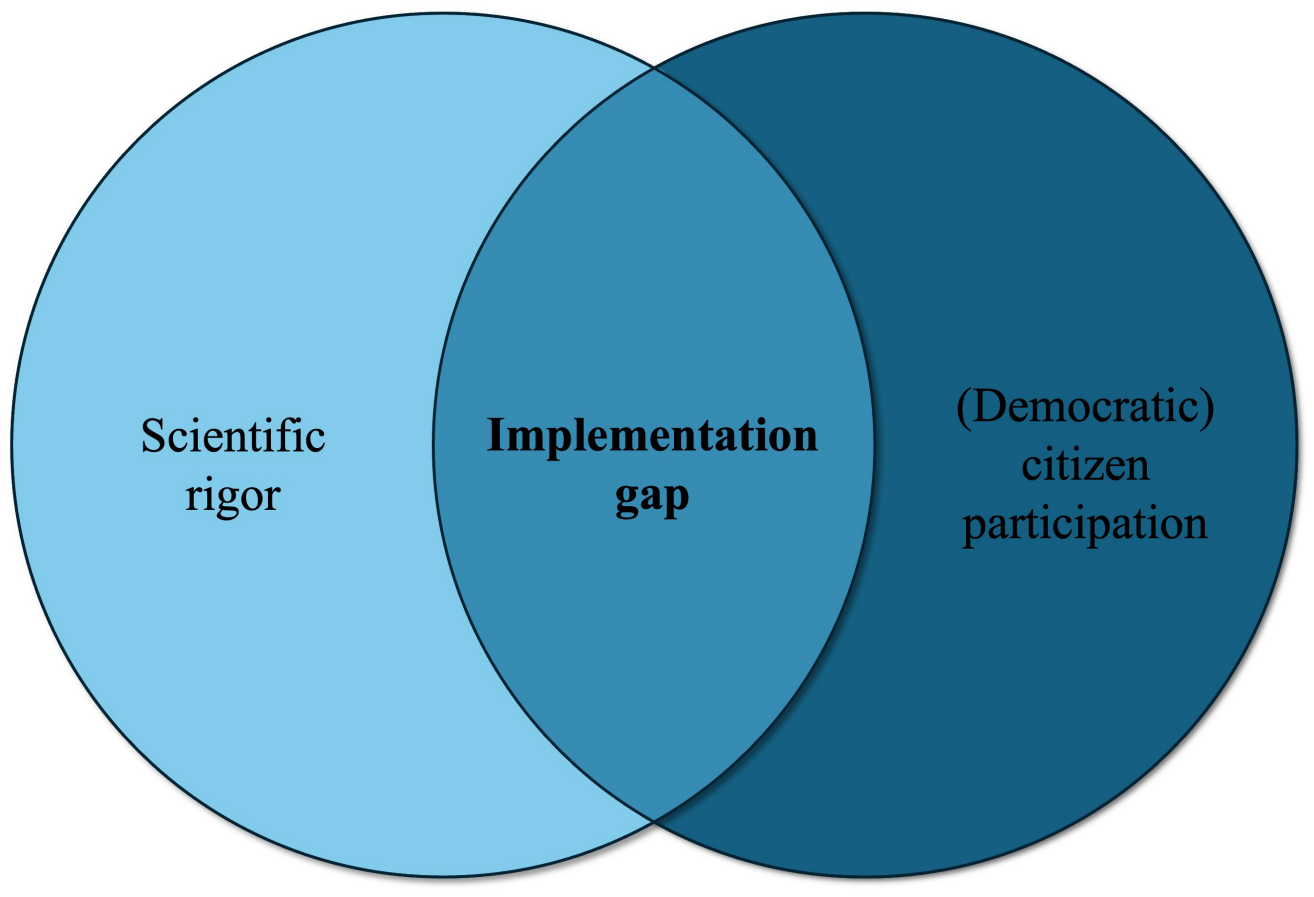
An implementation gap caused by a tension between upholding scientific rigor and democratic citizen participation.

Citizen science aims to produce credible knowledge while also generating citizen participation (6, 7). The European Citizen Science Association (8), for example, describes that “citizen science programs are evaluated for their scientific output, data quality, participant experience and wider societal or policy impact”. While quality criteria for rigor are well-established in the health sciences, there is no one set of criteria for defining quality participation, yet consensus exists that it should go beyond tokenistic forms of participation (9) – such as using citizens only for data collection – and instead be democratic (10, 11), as well as relational (4), critical (12), and transformative (9). Combining these aims creates a fundamental tension in positionality between neutrality and embedded engagement (12–14), thereby producing an implementation gap (see Figure 1). Rooted in a positivist epistemology, public health research prioritizes neutrality and impartiality as markers of scientific rigor, making citizen participation challenging due to concerns of bias. Consequently, citizen science projects often limit citizens' roles to the tokenistic tasks of data collection (“citizens as sensors”), while researchers retain control over defining research questions, interpreting data, and developing interventions (15). Doing otherwise is to risk losing epistemic credibility and being dismissed as an “academic lightweight” (16).

Some scholars argue that, in the final analysis, the implementation gap of citizen science cannot be fully closed without addressing fundamental epistemological debates. Without resolving these, citizen science may never reach its full potential and could risk being relegated back to the margins of scientific endeavor (17–20). In this article, we offer a different perspective. We argue that within a positivist epistemology, scientific rigor and participatory criteria may prove mutually beneficial through the participatory study of social-symbolic objects. The term social-symbolic object was first coined by the management and organizational scholars Lawrence and Phillips (21), to better understand how aspects of organizational life are constructed by collectively shared symbols, such as narratives, identities, assumptions, and routines. Given that these objects serve as determinants of health inequalities and are uniquely experienced by specific population groups, they may provide a foundation for research that is scientifically rigorous and genuinely participatory.

## Social-symbolic objects

Consider two examples of social-symbolic objects. In a recent study, Rodriguez et al. (22) examined the causal link between beliefs and loneliness and concluded that how people think about being alone shapes their experience of loneliness. The authors found that between 2020 and 2022, US newspaper headlines were 10 times more likely to frame being alone negatively than positively, and that exposure to these headlines influenced experiences of loneliness negatively. According to the authors, media and public health campaigns may inadvertently contribute to loneliness by negatively portraying the experience of being alone, thus swaying people's beliefs. In another study, Mennis et al. (23) found that in disadvantaged neighborhoods, high residential mobility—marked by frequent in- and out-migration and increasing diversity—undermined collective efficacy, a proxy for social cohesion. Mobility disrupts social ties, limiting residents' time and motivation to build relationships, which can negatively affect mental health, substance use, and child development. While relocation may benefit some individuals, widespread mobility can harm neighborhood wellbeing as a whole. In other words, programs fostering social mobility may unconsciously crowd out social cohesion.

These two studies, along with decades of research, indicate that people's health and wellbeing are shaped by beliefs (24, 25), and that these beliefs are often situated within collective narratives and frames (26–28). Popular discourses may become internalized by individuals as processes of cultural learning (29). Consider words such as “success”, “self-improvement”, and “mental illness” that are part of our everyday vocabulary, yet are value-laden and embedded in collective narratives and frames, reflecting deeper ideological assumptions about individual responsibility, normalcy, and failure. For instance, when individuals internalize the view that being “unsuccessful” is a personal failing, they may become more susceptible to mental illnesses such as depression (30). Also scientific and policy vocabularies play a role (31). Population descriptions in public health research such as “hard-to-reach”, “at-risk”, and “welfare dependent” are equally value-laden and may become internalized by individuals (32). Once these categories enter everyday language, they not only shape how individuals see themselves but also how communities are positioned in relation to broader systems of support, recognition, and intervention (33).

These examples may be referred to as social-symbolic objects, according to Lawrence and Phillips (21). These objects refer to combinations of discursive, relational, and material elements that constitute meaningful patterns in social systems. Social-symbolic objects are embedded in everyday practices and carry deep symbolic significance that structure how individuals interpret, experience, and navigate their everyday experiences. The origin of the term lies in a growing recognition of the importance of language in shaping social life. This “linguistic turn” reflects a growing recognition that language is not simply a tool for describing reality but also has a formative function in constituting social reality (34). Social-symbolic objects manifest within our worldviews as concepts (e.g., success, intelligence, property rights), ideas (e.g., idea of a career, of workdays and weekend days, of workdays being between 9 and 5), metaphors (e.g., viewing career as a ladder, history as one-directional as opposed to cyclical, jobs and partner seeking as market choices), and assumptions (e.g., ‘the ends justify the means', ‘division of labor produces economic prosperity'). By creating concepts and categories to describe the world, a social reality is created that shapes human life via institutional facts – facts by human agreement (35).

### Properties of social-symbolic objects

Social-symbolic objects have two key properties (21). First, they carry implicit assumptions that shape problem definitions and policy implications, which can either perpetuate or disrupt systemic health inequalities. For example, when campaigns reframed depression as a common and treatable condition rather than as a personal weakness, help-seeking behavior and treatment uptake improved (36). Yet, these assumptions are often deeply embedded in organizational routines. As Giddens (26) argues, the “vast bulk of the ‘stocks of knowledge' … is not directly accessible to the consciousness of actors,” but rather is embedded in the capacity to carry on within everyday social routines. Therefore, observing and understanding social-symbolic objects demands reflexivity and awareness of one's own social reality—qualities that citizen science can help cultivate through its cyclical and iterative research process. Furthermore, people experience social-symbolic objects differently depending on their personal experiences. In other words, social-symbolic objects are ‘observer-relative' (35). Thus, participatory research into social-symbolic objects may produce richer insights than non-participatory methods.

Second, social-symbolic objects have distributive effects and are therefore an important but underexplored determinant of health inequalities. Because these objects carry embedded meanings and normative assumptions, they influence who is perceived as deserving, capable, and legitimate within institutional contexts such as education, healthcare, and welfare. As a result, they shape access to resources and affect the life trajectories of individuals and groups. For example, the concept of meritocracy—the belief that success is earned through talent and effort—creates a ‘culture of winners and losers,' eroding the self-worth of individuals in low-status jobs (37). Similarly, defining poverty primarily as a lack of consumption may encourage people experiencing poverty to engage in status-driven consumption to gain social recognition. Yet, such consumption patterns may deepen their financial hardship, perpetuating cycles of poverty (38).

## Discussion

In this article, we have argued that the participatory study of social-symbolic objects not only accommodates the dual aims of rigor and participation, but can render them mutually reinforcing – unlike, for instance, the biomedical study of a physical disease. By contrast, lifestyle and mental diseases that contain social-symbolic properties such as personal responsibility, social identity, and belonging are shaped by lived experience and social narratives and therefore benefit from participatory approaches. In a similar vein, researchers seeking to foster meaningful participation may benefit from expanding their research focus to include social-symbolic objects into their research.

Some may question why social-symbolic objects require participatory methods rather than just interviews or ethnography – well-established and proven methods. Yet because social-symbolic objects are observer-relative, researchers without lived experience risk framing their studies in ways that are misaligned with participants' realities, asking inappropriate questions during data collection, or misinterpreting their findings. Collaborating with citizen scientists may yield more objectivity, drawing on a deeper lived experience of these objects (39). This “epistemic advantage” holds true not only for citizen scientists, but also professional scientists, who often draw on their own social identities, lived experiences, and political contexts in making key assumptions about their research (40). These personal starting points should not be confused with the resulting rigorous procedures used to separate facts from values. Participation can therefore be seen as a valuable addition to the researcher's methodological repertoire, as it operationalizes the insight from philosophy of science that observation is not neutral but shaped by the assumptions researchers bring to it – a point that remains underrecognized in mainstream public health research (41).

A critique on the other end of the spectrum is that this approach may still instrumentalize citizens for scientific purposes, rather than serving the interests of citizens more directly. We argue, however, that genuine participation in research may be a potential health intervention in itself – a promising avenue that requires more research. When citizens, through participation in research, learn first-hand the mechanisms by which narratives such as ‘being alone means being lonely' or ‘my life's circumstances are the result of personal failure' become a determinant of health, it not only increases awareness but may also foster a sense of personal control – bringing distant societal determinants into one's personal sphere of influence (42). It may also motivate citizens to initiate actions within their own communities. This aligns with insights from participatory action research that emphasize the importance of fostering intellectual self-capacity and self-assertion among marginalized groups to break free from oppressive narratives (43). Although oppression in this literature is often referred to in terms of political exploitation and power imbalances, they may also refer to the collective narratives highlighted above.

Looking ahead, the participatory study of social-symbolic objects may offer a promising approach to combining genuine participation with scientific rigor and credibility. Although many areas of public health research already engage with such objects, this work remains fragmented and lacks a shared terminology and a coherent agenda for learning about genuine participation. Advancing this approach will therefore require a more streamlined use of terms and concepts around genuine participation. We may also benefit from good examples of this approach. As a starting point, we point to a study by Raab, Knibbe, and Horstman (44) who co-produced several problem definitions and local matters of concern around the concept of neighborhood health through philosophy cafés, photovoice sessions, and park-based dialogues.
